# Rasch analysis holds no brief for the use of the Dermatology Life Quality Index (DLQI) in Chinese neurodermatitis patients

**DOI:** 10.1186/s12955-016-0419-5

**Published:** 2016-02-03

**Authors:** Yale Liu, Tian Li, Jingang An, Weihui Zeng, Shengxiang Xiao

**Affiliations:** Department of Dermatology, The Second Affiliated Hospital, School of Medicine, Xi’an Jiaotong University, 157 Xiwu Road, Xincheng District, Xi’an, Shaanxi Province 710004 People’s Republic of China; Department of Dermatology, Xijing Hospital, Fourth Military Medical University, Xi’an, Shaanxi 710032 People’s Republic of China

**Keywords:** Rasch analysis, Neurodermatitis, Dermatology Life Quality Index (DLQI)

## Abstract

**Background:**

The Dermatology Life Quality Index (DLQI) is the most widely used measure of health-related quality of life (HRQoL) associated with skin disease. Recently, the psychometric properties of the DLQI have caused some controversy because the instrument appears not to meet the requirements of modern test theory. The purpose of this study was to assess whether these psychometric issues also occur in Chinese patients with neurodermatitis.

**Methods:**

One hundred fifty consecutive outpatients (83 males and 67 females) seeking treatment for neurodermatitis were assessed for eligibility for this prospective study between July 1, 2011 and September 30, 2011. The DLQI and a demographic questionnaire were completed. One female participant who incompletely answered the DLQI was excluded. Data were analyzed using the Rasch model in order to obtain meaningful scores for the DLQI. Scale assessment included analysis of rating scale function, item fit to the Rasch model, aspects of person-response validity, unidimensionality, person-separation reliability, and differential item function.

**Results:**

The rating scale advanced monotonically for all items in the DLQI, but item 9 did not demonstrate acceptable goodness-of-fit (Infit MnSq values >1.3) to the Rasch model. The 10 items of the DLQI met the criteria for person-separation reliability (PSI = 2.38) and the first latent dimension (general QoL) accounted for 50.8 % of the variance; but the variance explained by the second dimension (7.1 %) exceeded the criterion of 5 %. There were also limitations related to person-response validity, because ≥ 5 % (18.1 %) of cases demonstrated unacceptable fit. There was no uniform differential item functioning.

**Conclusions:**

For neurodermatitis patients, the DLQI seems to have poor fit to the Rasch model; therefore, we recommend against using this instrument with neurodermatitis patients.

## Introduction

The Dermatology Life Quality Index (DLQI) [1] is a simple, practical questionnaire for routine clinical use and has been widely adopted as a patient-reported outcome measure in dermatology. It was developed more than 20 years ago according to the principles of classical test theory. Because of its reliability, validity and ease of use, it benefits both researchers and clinicians. It has been used for over 36 different skin conditions and translated into 55 languages [2]. However, the psychometric properties of the DLQI seems not to fulfill the requirements of modern test theory, e.g., Item Response Theory (IRT).

IRT is now seen as the new standard for developing and improving questionnaires [3, 4]. It has several advantages over classical test theory, particularly for measuring variables in the social sciences [5]. IRT parameters can be visualized by plotting item characteristic curves (ICCs). ICCs depict the probability of a response (endorsing a symptom) given the level of the underlying characteristic or disease state measured by the whole scale (such as severity of squamous cell carcinoma). In this study, ICCs are defined by difficulty and discrimination, that is, discrimination means how well an item can distinguish lower and higher quality of life while difficult means that results in a higher score (i.e., a lower quality of life), as described by Beth and colleagues [6], which govern the shape and position of S-curves. Recently, Rasch analysis, which is one specific application of IRT, has been applied to assess the DLQI [5]. The application of the DLQI for psoriasis, atopic dermatitis (AD) and hand eczema (HE) was criticized based on Rasch analysis [7–9]. The objective of the present study was to perform a psychometric test of the DLQI in a sample of neurodermatitis patients using the Rasch analysis.

Neurodermatitis, also called lichen simplex chronicus (LSC), is a disorder commonly encountered by dermatologists and caused by constant rubbing of the skin that clinically induces thickening and lichenification. Neck, elbow, ankles, vulva, eyelid, faces and even conjunctiva [10] can be affected. Although it is not life-threatening, LSC can result in psychosocial problems, and it has been suggested that patients who get it are more likely to suffer from depression, anxiety and other treatable psychological disorders [11]. It can also impair quality of life through sleep disturbance and sexual dysfunction [12]. All these data indicate that it is necessary to pay attention to the negative impact of neurodermatitis on patients’ quality of life (QoL).

In our previous study, an evaluation of the DLQI in LSC was conducted within the framework of classical test theory and demonstrated good feasibility and internal consistency [13]. However, to date, it appears that no studies available have used a Rasch analysis approach to examine the psychometric property of the DLQI in persons with LSC.

Therefore, the overall aim of our study was to use Rasch analysis to evaluate the DLQI in a sample of Chinese persons with LSC. In this study, we used Rasch analysis to test rating scale function, item fit to the Rasch model, aspects of person-response validity, unidimensionality, person-separation reliability, and differential item function (DIF)..

## Methods

### Main data collection

The psychometric properties of the DLQI were assessed in 150 consecutive outpatients seeking treatment for LSC in the Department of Dermatology, The Second Affiliated Hospital of Xi’an Jiaotong University between July 1, 2011 and September 30, 2011. Demographic data and disease-related characteristics were collected. All subjects gave informed consent prior to participation. Patients less than 18 years old or having any other skin/systemic disease or mental disorder were excluded. All participants provided written informed consent. The study was approved by the ethics committee of the hospital.

### Dermatology Life Quality Index (DLQI)

The DLQI was first translated into Chinese in 2004 by Zhao et al., with authorization from its creator, Andrew Finlay, and in collaboration with the New Territories Centre for Health Education, Shatin, Hong Kong [14]. The DLQI is a 10-item self-reported questionnaire that assesses the impact of skin disease on the patient over the last week [1]. Ten questions concerning symptoms, feelings, daily activities, leisure, sport, work or school, personal relationships and treatment. Nine items have four response options: “Very much (0),” “A lot (1),” “A little (2),” and “Not at all (3).” Item 7 first asks whether working or studying has been prevented and then to what degree the skin condition has been a problem for working or studying (“A lot”,“A little”, “Not at all”). Eight items also have a “Not relevant” option scored “0,” which indicates no problem. Individual item scores are summed to give a total score that ranges between 0 and 30. Higher scores indicate lower QoL.

### Dermatology Index of Disease Severity (DIDS)

The DIDS is an efficient instrument for staging the severity of illness in inflammatory cutaneous diseases [15]. It measures two elements [16]: (i) the percentage of body surface area involvement, which is determined by the ‘1–2–3–4 rule’, in which the scalp ⁄neck ⁄face are assessed as 10 %, arms as 20 %, entire trunk as 30 % and legs plus buttock as 40 %; and (ii) functional limitations (i.e., difficulty in holding a pen or walking for up to 30 m), which are determined by the healthcare worker who performs an objective evaluation during the consultation. The DIDS categorizes 5 stages of active clinical disease: 0, none; I, minimal; II, mild; III, moderate; and IV, severe.

### Rasch analysis

Detailed explanations of the required analyses have been published previously [5, 17, 18]. The same procedures of Rasch analysis were followed for our sample. Minifac Version No. 3.67.1 softwarewas applied to the DLQI data.

#### Unidimensionality

Assuming a unidimensional scale means that all test items (in this case, questionnaire items) assess a single underlying construct [19]. To determine whether DLQI measures a single underlying construct and to minimize the risk of additional explanatory factors in the measures generated, the unidimensionality of the items was examined and a principal component analysis was performed. Two criteria needed to be met: 1) at least 50 % of the total variance explained by the first latent dimension, and 2) < 5 % of the remaining variance explained by any additional dimension, after removal of the first latent dimension [5].

#### Fit to the Rasch model

Rasch model fit is primarily indicated by non-significant fit statistics, indicating that the scale does not deviate from model expectations [18]. The fit of the data to the Rasch model is the desired outcome of the analysis [20]. Item goodness-of-fit statistics are used to test whether the individual item fits the Rasch model. Item fit was investigated using goodness-of-fit statistics to generate mean square (MnSq) residuals for all items and persons. These values indicate the degree of match of actual responses with the DLQI. The criterion for evaluating the fit to the Rasch model was to accept fit index Infit MnSq values between 0.7 and 1.3 logits [21].

#### Rating scale functioning

The functioning of the DLQI rating scale was evaluated according to the criteria made by Bonsaksen et al. [5] to ensure the rating scale functions consistently across items. A criterion of Outfit MnSq values < 2.0 for each item was set for response category calibration.

#### Person response validity

To assess how well the individual responses matched expected responses from the Rasch model, person-response validity was investigated using goodness-of-fit statistics to generate MnSq residuals and standardized z-values for persons. The criteria for acceptable goodness-of-fit values were Infit MnSq < 1.5, z ≤ 2.0 and ≤ 5 % of sample fails to demonstrate acceptable goodness-of-fit values.

#### Person-separation reliability

To further determine whether the DLQI could distinguish distinct levels of QoL in the sample tested, person-separation reliability was assessed. Distinguishing at least three levels of QoL (high, medium, and low) requires a person separation index (PSI) of at least 2.0.

#### DIF

Items that do not yield the same item response function for two or more groups display DIF and violate the requirement of unidimensionality [8]. To ensure that responses to individual items were not differentially affected by external factors, including age, sex and disease severity, DIF analyses were performed. If the p-value of the analysis of variance of standardized residuals across a factor was less than 0.05, an item was defined as having significant DIF, which suggests that an item lacks generalizability in populations that span different patient groups [22]. Although a Bonferroni correction yielding an alpha value of 0.01 is commonly used [23], we also report results with p < 0.05 to more conservatively evaluate the likelihood of item bias.

## Results

### Sample characteristics

A total of 150 individuals participated; data from 149 (99.3 %) were analyzed. The one participant whose data were excluded failed to answer 7 of the 10 DLQI items. The socio-demographic characteristics of the 149 participants included in the analysis are presented in Table 1. There was no significant difference in DLQI scores with respect to age, sex, employment status, education or location, but differences were found according to disease severity, where more severe disease was associated with higher DLQI scores (*p* < 0.001).

**Table 1:** Demographic characteristics of the sample and DLQI scores (*N* = 149)

	Demographic characteristic	DLQI scores mean (SD)	*P*-value
Sex n (%)	Male 83 (55.7)	10.17 (6.75)	0.073
	Female 66 (44.3)	8.24 (5.80)	
Age n (%)	18–35 78 (52.3)	8.75 (6.85)	0.259
	36–83 71 (47.7)	9.95 (5.80)	
Disease severity n (%)	I 31 (20.81 %)	7.76 (5.88)	<0.001
	II 108 (72.48 %)	11.61 (6.02)	
	III 10 (6.71 %)	17.40 (6.86)	
Employment status n (%)	Employed 89 (59.7)	9.30 (6.75)	0.429
	Unemployed 17 (11.4)	7.47 (4.30)	
	Student 21 (14.1)	9.19 (6.76)	
	Retired 22 (14.8)	10.91 (5.82)	
Education n (%)	Primary 7 (4.7)	9.67 (5.31)	0.938
	Secondary 57 (38.3)	9.38 (6.53)	
	>Secondary 85 (57.0)	9.16 (6.70)	
Location n (%)	Urban 41 (27.5)	11.37 (6.02)	0.621
	Rural 108 (72.5)	8.52 (5.38)	

For Rasch analysis, sample size requirements are influenced by scale targeting. For a scale that is well targeted (i.e., 40–60 % endorsement rates for dichotomous items), a sample size of 108 will give accurate estimates of person and item locations (99 % confidence of locations being within 0.5 logits) [18].

As shown in Table 1, there was no patient in stage 0 or IV. Thirty-one (20.81 %) patients were in stage I, 108 (72.48 %) in stage II, and 10 (6.71 %) in stage III. Patients with lower disease stage reported a significantly lower DLQI scores.

### Unidimensionality

We found DLQI did not measure a single underlying construct. However, dimension 1 accounted for 50.8 % of the variance in DLQI score in this setting, which met the criterion for the first dimension. The secondary latent dimension explained an additional 7.1 % of the variance, which was slightly higher than the expected 5 %. Therefore, evidence of unidimensionality is mixed in the 10-item DLQI.

### Fit to the Rasch model

**Fig. 1:**
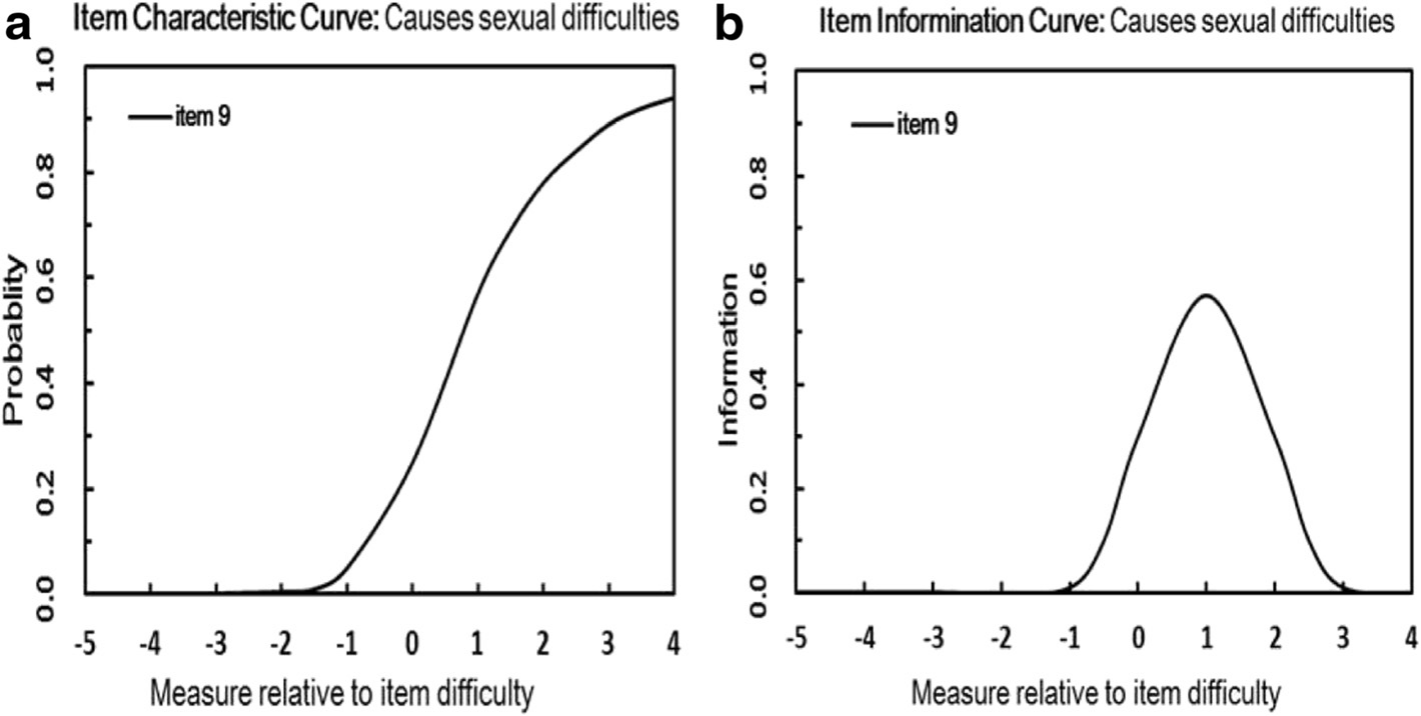
Item characteristic curve and item information curve for item 9. The curves represent the item as high difficulty − low discrimination because it is more likely to be endorsed at high levels of severity. However, it is relatively imprecise in measuring individual differences among respondents. X-axis - the latent trait of HRQoL. Y-axis - the probability of participant response. **a** Item Character Curve, **b** Item Information Curve

Findings from assessing the fit of the DLQI to the Rasch model are presented in Table 2. Analysis of content validity of the 10 DLQI items revealed that item 9 (Infit MnSq values > 1.3) did not demonstrate acceptable goodness-of-fit to the Rasch model, meaning that the participants’ scores on this particular item were inconsistent with their overall response patterns. For illustrative purposes, the ICC and item information curve for item 9 is presented in Fig. 1. As described earlier, ICCs graphically depict the probability of the response to an item, given a particular level of the underlying state being measured. Item information curve for item 9 demonstrates decreased discrimination at the moderate to upper levels of severity in the LSC patients.

**Table 2:** Items, measures, and item statistics of the 10-item version of the DLQI

Item No.	Item description	Item measure (Location value^a^)	Item fit statistics (Infit MnSq)
1	Itchiness, soreness, pain, or stinging	0.9	1.10
2	Embarrassment/self-consciousness	−0.5	0.94
3	Interferes with shopping/looking after home/garden	−1.2	0.84
4	Influence choice of clothing	0.3	1.03
5	Affects social/leisure activities	−0.5	0.93
6	Affects ability to play sports	−0.5	0.93
7	Prevents working/studying	−1.8	0.79
8	Creates problems with partner/close friends/relatives	0.4	1.05
9^b^	Causes sexual difficulties	3.4	1.47
10	Problems with treatment	0.1	1.01

^a^ The lowest/highest value indicates that the corresponding item is assessing the mildest/most severe impairment

^b^ This item showed critical misfit according to the Rasch model and had to be removed during the analysis

### Rating scale functioning

**Fig. 2:**
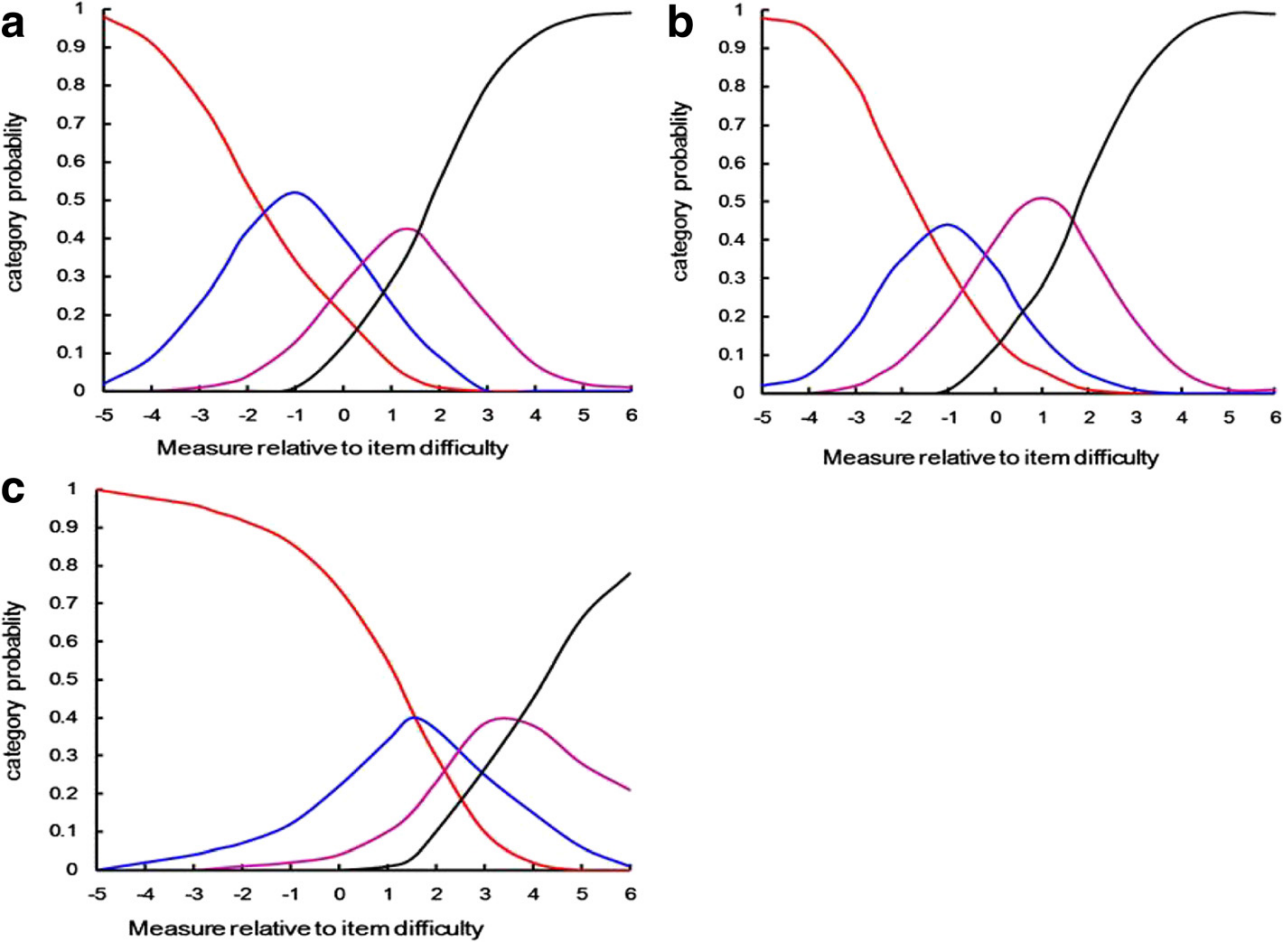
Rasch model category probability curves for all DLQI items together, showing the likelihood that a participant with a particular subjective severity level will select a category. The x-axis represents the latent trait of HRQoL, and the y-axis represents the probability of response category being selected. Curve a for item 2,3,5,6,7, and curve b for item 1,4,8,10 and curve c for item 9 are shown respectively. For any given point along this scale, the category most likely to be chosen by a participant is shown by the category curve with the highest probability. Red: not at all/not relevant; blue:a little; pink: a lot; black:very much

Examining the distribution of responses revealed irregularities in the use of the response categories: the response “Not at all/Not relevant” had been used in 39 % of responses and the response “A lot” in only 9 % (Table 3). However, no step disordering was found in the scale category measures, which indicates that the scale categories worked as intended. The Outfit MnSq was less than 2.0, indicating randomness in the choice of categories [17]. Hence all the responses in these categories are not a threat to the validity of the DLQI. Figure 2 shows the category probability curves for 10 items separately, which illustrate the probability that each of the four response categories is chosen throughout the range of HRQoL. The probability of responding to different categories changes along the logit scale; this provides further evidence that the response scale produced ordered thresholds. As item 2,3,5,6,7 have the same similar question type, we analysed them together, the same as item 1,4,8,10. But for item 9, it is more likely to be endorsed at high levels of severity, which is consistent with the result of ICC for 9 item.

**Table 3:** Functionality of the rating scale

Scale category	Frequency (%)	Average category measure	Outfit MnSq
Not at all/ Not relevant	573 (39)	−2.33	1.1
A little	539 (37)	−1.11	0.9
A lot	222 (15)	0.30	0.7
Very much	126 (9)	1.17	1.2

### Preson-response validity

Of the 149 DLQI surveys analyzed, 27 (18.1 %) failed to demonstrate acceptable goodness-of-fit to the Rasch model in the 10-item version, indicating that the response patterns of these persons were unlikely given their underlying level of QoL. There were no systematic demographic differences between respondents with and without misfit. It was concluded that the DLQI demonstrated a somewhat higher level of misfit among participants than expected.

**Fig. 3:**
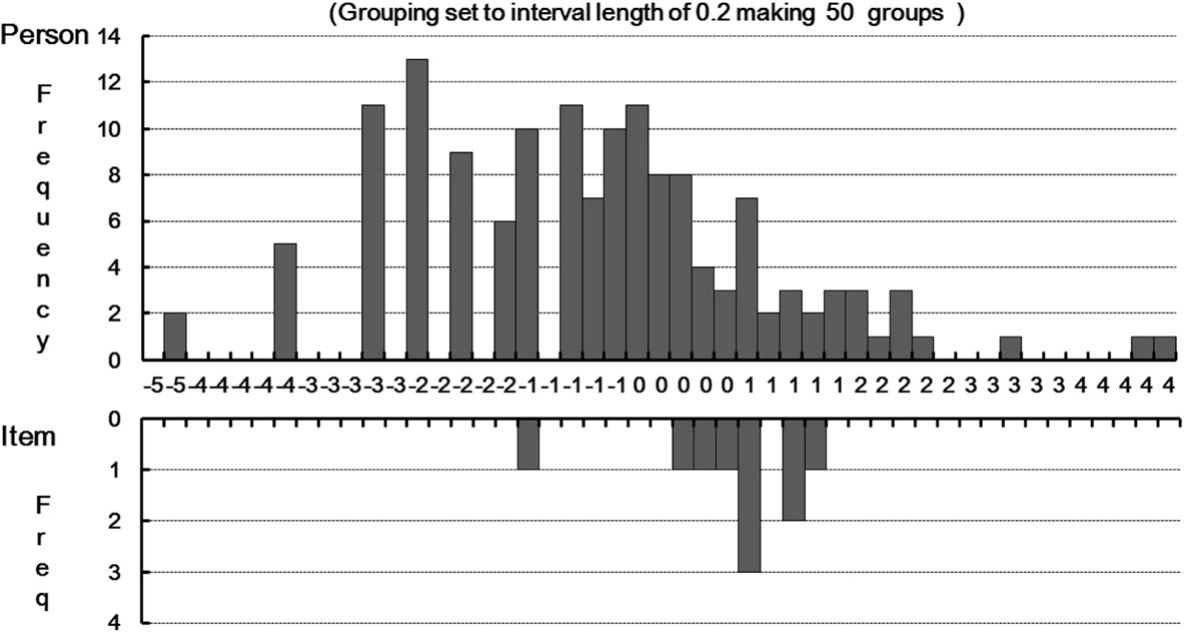
Person and item threshold distribution for the DLQI. Upper figure: the distribution of persons across the logit scale of DLQI impairment. Lower figure: the distribution of the item thresholds on the same scale

To monitor the targeting of the DLQI in relation to the current sample, the number of participants with maximum and minimum scores across the different DLQI-item solutions was evaluated. Four participants (2.7 %) were outside the maximum range of the DLQI 10-item version (two above and two below the maximum range). The distribution of the sample with respect to the item thresholds is presented in Fig. 3.

### Person-separation reliability

To examine the DLQI in terms of its ability to separate persons into groups based on different levels of QoL, person-separation reliability was analyzed. The PSI was 2.38 for the10-item DLQI, indicating that it can detect three statistically distinct groups of participants within the sample. The distributions of persons and DLQI items (and each item threshold per response category) for the ten items are presented in Fig. 3. The lower part of Fig. 3 shows the distribution of the item thresholds on the same scale. The graphs show that the thresholds are clustered in the middle of the scale (−0.6 to 1.2) and are therefore redundant in this area. In contrast, items assessing severe impairment in DLQI are missing (see Fig. 3 values < −0.6, lower graph), and > 75 % of the investigated LSC population fall within this area (see Fig. 3 values < −0.6, upper graph).

### DIF

Table 4 shows how the DLQI rating scales functioned across age, sex, location, education level, and severity of illness. The DLQI items functioned similarly in relation to participant sex and location. However, items 2 and 4 functioned differently by age group, and items 4 and 8 functioned differently by severity of illness. Item 4 functioned differently in relation to educational level. When the significance level was adjusted for the number of comparisons (*p* < 0.01) none of the DIFs were significant in the 10-item version.

**Table 4:** Differential item functioning in the original 10-item DLQI version

Differential item functioning	Results (Original 10-item DLQI)
Age	Item 2: more agreement among people < 35 (*p* = .028)
	Item 4: more agreement among people < 35 (*p* = .015)
Sex	No DIF
Location	No DIF
Education	Item 4: more agreement for higher education (*p* = .038)
Severity of illness	Item 4: more agreement for patients with more severe condition (*p* = .027)
	Item 8: more agreement for patients with more severe condition (*p* = .041)

The criterion for differential item function was a Mantel–Haenszel statistic with *p* < 0.01 after Bonferroni correction [21]. Using an uncorrected *p* < 0.05 is not common, but minimizes the risk of underestimating item bias. After Bonferroni correction, no differential item functioning was observed

## Discussion

This study is, to the best of our knowledge, the first to use Rasch analysis to examine the psychometric properties of the DLQI in a sample of patients with LSC. Psychometric deficiencies that have previously been reported for the DLQI’s use with other disorders were also found in Chinese patients with LSC.

In previous studies, Rasch analysis was applied to the DLQI data to study its psychometric properties in patients with psoriasis, AD and HE. In psoriasis and AD patients, inadequate measurement properties were observed [7]. One study revealed disordered thresholds and DIF for data from psoriasis patients across different cultures [9], and another detected similar problems with data from patients with HE [8]. Our study documented psychometric problems with LSC patients.

None of the response categories pose a threat to the validity of the DLQI. Good separation ability of the 10-item version (PSI 2.38) indicates that the items may reliably identify persons with higher QoL and well-being. The first latent dimension (general QoL) accounted for 50.8 % of the variance in the 10-item DLQI, but item 9 demonstrated poor fit to the Rasch model. The variance explained by the second dimension (7.1 %) exceeded the criterion of 5 %, suggesting the possibility of a minor second dimension. There were also limitations related to person-response validity, with ≥ 5 % (18.1 %) of cases in the 10-item DLQI demonstrating unacceptable fit to the Rasch model. Figure 1 shows that the DLQI has a poor spread of thresholds, indicating a limitingmeasurement range.

Item 9 demonstrated misfit to the Rasch model. This may indicate a general misfit of the item across sample populations, and not a particular misfit among persons with LSC, given that a study on HE patients found similar problems with this item [8]. Item misfit essentially indicates that the respondents rated this item inconsistently in relation to their overall response pattern. For item 9 the misfit may be explained by a difference between Asian and Western cultures. Discussing sexual activity is a cultural taboo, and body contact is not considered acceptable in everyday interactions between men and women. Recently in Chinese society, notions of sexual activities have changed considerably; however, it is still considered too private an issue to discuss frankly, even with friends or family. Therefore, the forthrightness of respondents’ answers remains unknown. It is possible, however, that the LSC is not severe enough (especially in stage II) to influence respondents’ sexual lives, or that most of the LSC lesions were not in the anogenital region. Ideally, we would have analyzed the effect of lesion locations on sexual life. Of course, it would be a disservice to eliminate an item that had particular relevance to a segment of the LSC population [24, 25].

Removing item 9 could also make the total score of the remaining items difficult to compare with previous research results. Therefore, if future studies with participants with LSC use a modified version of the DLQI, the scores should be adjusted to correct for the reduced number of items. However, comparisons with 10-item scores, in particular to shorter scales composed of different items, should be made with caution as there are still controversies regarding comparative outcomes [9, 26].

Our study also showed lower DLQI levels for our participants than have been reported previously with psoriasis population samples [7, 27], which is consistent with our previous study [13]. This makes sense because severe itching is a prominent feature of LSC but is rarely a concern for psoriasis patients. Consistent with our finding, Reich and colleagues recently found itching in 89.2 % of psoriatic patients [28], and the presence and intensity of itching did not depend on age, sex, type of psoriasis, duration of disease or disease severity [29]. In addition, LSC patients had more sexual problems than did their counterparts with psoriasis [24]. Further researches are needed to examine the observations and test them with Rasch analysis.

In contrast, age, education and severity of illness may be related to QoL. In a sample of patients younger than 35 years, QoL was substantially lower (mean DQLI = 8.75) than in a sample of patients over 35 (mean DQLI = 9.95). The difference was discussed in light of psychological factor theory [12], that is, the younger, the more anxious and therefore the more severe and persistent LSC. For patients with a higher education level, LSC influenced their ability to dress appropriately. Thus, the lower levels of QoL in this group may be partly attributable to their attention to appearance, which might be rooted in unstable situations and the stress they may experience during the process of job change. This comparison may indirectly speak to a larger impact of stress, rather than age, on QoL. Dressing up and having close relationships were both affected in patients with severe LSC; it is to be expected that severe itching and lichenoid skin would affect such aspects of QoL. In addition, there is no evidence of DIF for sex in our study, which is different from previous reports [25, 30] . When the effect on QoL of a specific entity is being considered, the larger sample, the more representative the result will be. As for sex, its influence on QoL displays no common pattern, although sex effects have been reported previously in patients with HE [31] and psoriasis [32]. During the investigation, many patients reported fear of the effects of diet on LSC. This factor is not included in the DLQI because of cross-cultural differences, although it may affect QoL. In addition, many items are ambiguous; that is, one item may include more than one idea. For example, item 5 asks how much the patient’s LSC has affected any social or leisure activities. With such an item, one patient may respond to the “social” aspect of the question while another considers the “leisure” aspect; this becomes problematic for the measure if different parts of the question represent different levels of impact. In addition, there are many manual workers in China, and labor is exercise for them. Therefore, it is difficult for them to evaluate the influence of a skin disease on sports activities, as described in item 6. Item 6 exceeded our expectations for fit to the Rasch model in our analysis.

### Strengths and limitations

This is the first study to report on Rasch analysis of the DLQI in LSC. The study was based on a sample of patients with a high participation rate (99.3 %) and relatively little missing data, thereby minimizing the likelihood of bias. The total sample of 149 provided a sufficient number for Rasch parameter estimates as it was large enough to give 99 % confidence that the reported estimates fall into a range of +/− 0.5 logits. Furthermore, evaluating the psychometric properties of the DLQI allows researchers and clinicians to decide whether they can benefit from it. In terms of limitations, the study was conducted in a single hospital and respondents were predominantly of Chinese cultural background. Despite a number of studies that have demonstrated sexual dysfunction associated with LSC, our study found that items about sexual difficulties have poor fit to the Rasch model, possibly because of dimensions of sexuality in the Chinese polutation. Research with other cultural populations may show quite different experiences of sexuality in respondents with LSC. We did not analyse the impact of lesion locations on QoL. Finally, this study only illustrated the use of the DLQI with LSC. The DLQI is also used with several other dermatological conditions, and further research could determine whether the scale is appropriate for use with those conditions.

## Conclusions

The DLQI was one of the first disease-specific HRQoL instruments to be developed in dermatology and has contributed greatly to our understanding of the patient’s perspective on LSC. However, this study suggests that the instrument is not well suited to measure disease impact among LSC patients in China. The DLQI is multidimensional, has limitations in person-response validity, and has two items that displayed non-uniform DIF. Also, the generalizability of the total scale and the underlying structure of the subscales could not be confirmed. The psychometric properties of instruments designed to assess the overall impact of a disease and its treatments on patients’ lives should be thoroughly tested in populations that vary culturally, demographically, and in disease severity before they are recognized as valid HRQoL assessment tools.

In sum, for LSC patients, the DLQI seems to have poor fit to the Rasch model in this population. As such, DLQI is not an approriate measure of QoL in Chinese patients with LSC.

## Abbreviations

AD: Atopic dermatitis
DIDS: Dermatology Index of Disease Severity
DIF: Differential item function
DLQI: Dermatology Life Quality Index
HE: Hand eczema
HRQoL: Health-related quality of life
ICCs: Item characteristic curves
IRT: Item Response Theory
LSC: Lichen simplex chronicus
MnSq: Generate mean square
PSI: Person separation index
QoL: Quality of life
